# Whole Grain Intakes Are Associated with Healthcare Cost Savings Following Reductions in Risk of Colorectal Cancer and Total Cancer Mortality in Australia: A Cost-of-Illness Model

**DOI:** 10.3390/nu13092982

**Published:** 2021-08-27

**Authors:** Mohammad M. H. Abdullah, Jaimee Hughes, Sara Grafenauer

**Affiliations:** 1 Department of Food Science and Nutrition, Kuwait University, Kuwait City 10002, Kuwait; 2 Grains & Legumes Nutrition Council, 1 Rivett Rd, North Ryde 2113, Australia; j.hughes@glnc.org.au (J.H.); sarag@glnc.org.au (S.G.); 3 School of Medicine, University of Wollongong, Northfields Avenue, Wollongong 2522, Australia

**Keywords:** colorectal, cancer, whole grain, healthcare cost, cost saving analysis, nutrition economics

## Abstract

Whole grain consumption has been associated with the reduced risk of several chronic diseases with significant healthcare monetary burden, including cancer. Colorectal cancer (CRC) is one of the most common cancers globally, with the highest rates reported in Australia. Three servings of whole grains provide a 15% reduction in total cancer and 17% reduction in CRC risk; however, 70% of Australians fall short of this level of intake. The aim of this study was to assess the potential savings in healthcare costs associated with reductions in the relative risk of CRC and total cancer mortality following the whole grain Daily Target Intake (DTI) of 48 g in Australia. A three-step cost-of-illness analysis was conducted using input parameters from: (1) estimates of current and targeted whole grain intakes among proportions (5%, 15%, 50%, and 100%) of the Australian adult (≥20 years) population; (2) estimates of reductions in relative risk (with 95% confidence intervals) of CRC and total cancer mortality associated with specific whole grain intake from meta-analysis studies; and (3) estimates of annual healthcare costs of CRC and all cancers from disease expenditure national databases. A very pessimistic (5% of population) through to universal (100% of population) adoption of the recommended DTI in Australia were shown to potentially yield savings in annual healthcare costs equal to AUD 1.9 (95% CI 1.2–2.4) to AUD 37.2 (95% CI 24.1–48.1) million for CRC and AUD 20.3 (95% CI 12.2–27.0) to AUD 405.1 (95% CI 243.1–540.1) million for total cancers. As treatment costs for CRC and other cancers are increasing, and dietary measures exchanging whole grains for refined grains are not cost preclusive nor does the approach increase energy intake, there is an opportunity to facilitate cost-savings along with reductions in disease for Australia. These results suggest specific benefits of encouraging Australians to swap refined grains for whole grains, with greater overall adherence to suggestions in dietary guidelines.

## 1. Introduction

Cancer care represents a leading burden of disease globally and accounts for 19% of the total disease burden in Australia [1]. Colorectal cancer (CRC) is the third most common in terms of new cases of cancers globally (1.93 million globally), after breast and lung cancers, and second most common in terms of causes of cancer death (935,000 deaths) in 2020, after lung cancer deaths [2]. Aligned with this, there is a significant healthcare monetary burden attributable to cancer including the direct and indirect healthcare costs and income losses. Furthermore, both Australia and New Zealand have CRC rates ahead of other countries [3]. Based on 2015/2016 data, cancer accounted for an estimated AUD 9.7 billion in diagnosing and treating cancer or just over 8.6% of all direct health expenditure, including AUD 876 million in costs for CRC including AUD 56 million on the National Bowel Screening program [4]. Although the mean age of diagnosis for CRC is 69 years, there is an increased risk after the age of 50 years, and more recent data suggests the incidence of an early-onset CRC under 40 years of age has emerged over the last two decades [5].

Nutrition, family history, body weight, alcohol intake, smoking, and sedentary lifestyles have all been implicated in the complex etiology of cancers, including CRC. Of all types of cancer though, there are a greater number of dietary factors influencing CRC. The World Cancer Research Fund and the American Institute for Cancer Research [6] noted in the Continuous Update Project, strong evidence that whole grain intake, and foods containing dietary fiber, dairy products, and calcium supplements (>200 mg/day), were protective against CRC and that red and processed meats, alcoholic drinks, excessive energy intake, and fast foods were associated with a higher risk of CRC. Lower-level evidence suggests that consuming foods containing vitamin C and consuming fish, multivitamins, and vitamin D supplements might decrease CRC risk, whereas low intake of non-starchy vegetables, low fruit, and foods containing heme iron might increase the risk. Physical activity was found to protect against colon cancer only [6].

A recent systematic review and meta-analyses of observational studies [7] identified 17 publications, involving 54 distinct meta-analyses for whole grains and five for refined grains, and reported significantly lower risk of total cancer and site-specific cancers in both “dose-response” and “highest vs. lowest” whole grain intake analyses [7]. Specifically, six studies examined CRC [8,9,10,11,12,13], four of which included dose-response data and suggested a 15–17% reduction in CRC risk for each 90 g/day intake of whole grains [7]. On the other hand, seven dose-response analyses indicated that 50–90 g/day intake of whole grains was associated with a 9–20% lower total cancer mortality risk. In these studies, the weight of the whole grain food was utilized, with 90 g (or three servings) providing approximately 48 g of whole grain. This difference in calculation of whole grain has been clarified by Ross et al. [14] and utilized in previous research of this nature [15].

Of concern is the greater increase in early-onset CRC in recent years. A re-analysis of the National Nutrition Physical Activity Survey (NNPAS) indicated that the median daily intake of whole grains was 21 g for adults (19–85 years) and 17 g for children/adolescents (2–18 years) [16]. Less than one third of children/adolescents (30%) did not consume whole grains on the day of the survey and consumption was lowest for the 14–18 years age group at 8.7 g/d [16], far short of the Daily Target Intake (DTI) of 48 g for Australians aged 9 years and older.

The suggested mechanism for the protection delivered by whole grains has been described previously [17]. Firstly, the intrinsic components of dietary fiber, resistant starch, oligosaccharides, and fermentable carbohydrates are thought to be protective through increasing fecal bulk and the production of short-chain fatty acids. Secondly, the micronutrient and antioxidant profile, with many of the vitamins and minerals having antioxidant effects. Thirdly, through mediation of the glucose response following consumption.

Over the past decade, numerous studies in the rapidly emerging field of nutrition economics have revealed substantial healthcare and related cost savings following healthy dietary behaviors among populations, accompanied by increasing scientific and public interests. Recently, a cost-of-illness analysis by our group [15] estimated an annual saving equal to AUD 1.4 billion in the combined type 2 diabetes (T2DM) and cardiovascular disease (CVD) related healthcare and lost productivity costs with higher whole grain intakes among Australian adults. Following a similar approach, the aim of the present study was to assess the potential savings in healthcare costs associated with reductions in the relative risk of CRC and total cancer mortality following the DTI 48 g for whole grains in Australia.

## 2. Materials and Methods

### 2.1. Study Design

In a conceptual framework model of input parameters derived from the relevant medical literature, a national nutrition survey, and a disease expenditure database in Australia, a three-step cost-of-illness analysis was developed based on: (1) current and targeted whole grain intake among estimates of proportions of the Australian adult population (≥20 years); (2) estimates of percent reductions in relative risk (with 95% confidence intervals) of CRC and total cancers mortality associated with whole grain intake; and (3) annual healthcare costs of CRC and all cancers in Australia. As previously modeled [15], a sensitivity analysis of four scenarios (very pessimistic, pessimistic, optimistic, and universal) was created to explore the impact of uncertainty, resulting in a range of assumptions withing each step.

The present analysis was conducted to reflect the reductions in cancer-related healthcare costs when the current intake of whole grains, as reported by the 2011-13 Australian Health Survey [16], were increased to the DTI level of 48 g/day for adults [18,19,20]. This is the cut-off value that policy makers, dietitians, and other healthcare providers typically use as guidelines in Australia. Table 1 summarizes the input parameters, and details around the analysis are discussed below.

### 2.2. Step 1: Estimation of Current and Targeted Whole Grain Intakes among Proportions of Prospective Consumers

Insights around the consumer perception and dietary behavior are an integral part of any public health nutrition model. The first step of the present analysis made assumptions around the adult population that would adopt the targeted level of whole grains in Australia. Based on the dietary intake data from the 2011–2012 NNPAS (*n* = 12,153) [16], the current median whole grain intake of 21 g/day for adults (19–85 years) was compared to an established DTI of 48 g [18,19,20] and a calculation was built on estimates of proportions of Australian adults (20 y and over) who are likely to increase their intake of whole grains by the “gap” amount of 27 g daily.

As previously described [15], in applying the sensitivity analysis to this step, the very pessimistic and pessimistic scenarios were set to predict healthcare cost savings when 5% and 15% of Australian adults increase their whole grain intakes to the DTI of 48 g over the practical short term (0–4 years) and short-to-medium term (5–9 years), respectively. The optimistic scenario assumed that 50% of Australian adults would increase their intake of whole grains, denoting a medium-to-long term (10–14 years) pragmatic estimate of potential savings. Lastly, the universal scenario was used to assume a 100% uptake rate, i.e., all adult consumers would increase their whole grain intake from the current median to target levels, and to represent a best-case long term (15–19 years) estimate of potential savings. It is important to note that 73% of all Australians >9 years of age consumed less than the 48 g DTI, and 30% were considered non-consumers of whole grain [16].

### 2.3. Step 2: Evaluation of the Percent Reduction in Risk of Colorectal Cancer and Total Cancer Mortality

Numerous observational studies, and meta-analyses thereof, have associated whole grains with a reduced risk of total and site-specific cancers. Following a key word search and identification of the relevant English-language literature, the second step of this analysis estimated the percent reduction in relative risk of CRC and total cancer mortality, separately, corresponding to the mean gap between the current intake and DTI of whole grains in Australia, by employing data from two systematic reviews and dose–response meta-analyses of prospective studies.

The first of these meta-analyses included 25 studies (6 reporting on whole grain intake and CRC risk, with a total of 7941 cases among 774,806 participants) and suggested a summary relative risk (RR) per 3 servings (using 30 g of food as a serving size) per day, equal to 0.83 (95% CI 0.78–0.89; I^2^ = 18%, P_heterogeneity_ = 0.30) [9]. A relative risk of 0.83 indicates a 17% relative risk reduction for CRC. I^2^ is the amount of total variation that is explained by variation between studies and P_heterogeneity_ is used to determine whether significant heterogeneity exists. The second meta-analysis included 45 studies (six reporting on whole grain intake and risk of total cancer, including 34,346 deaths from cancer among 640,065 participants) and suggested a summary RR per 90 g/day (equivalent to 3 servings) of 0.85 (95% CI 0.80–0.91; I^2^ = 37%, P_heterogeneity_ = 0.16) [21]. A relative risk of 0.85 indicates a 15% relative risk reduction for total cancer. Building on the RRs for CRC and total cancer risk per 90 g of whole grain per day, while assuming a linear relationship, an RR reduction per 27 g whole grain intake was calculated (as shown in Table 1) for use in the final step of the analysis. In the present analysis, 30 g of whole grain “product” was assumed to be equivalent to 16 g of whole grain “content”, with three servings being in line with the recommended DTI of 48 g. This conversion was recommended by the recent work of Ross et al. [14] and utilized in another analysis in the US by Murphy and Schmier [22].

### 2.4. Step 3: Calculation of the Potential Savings in Cancer-Related Direct Healthcare Costs

The third and final step of the present analysis imputed the annual savings in costs of healthcare-related services that could follow the estimated percent reductions in risk of CRC and total cancer mortality, separately, with the recommended increase in whole grain intake. Similar to our recent analysis [15], the 2015–2016 estimates of cancer-related direct health expenditure by the Australian Institute of Health and Welfare (AIHW) [23] were first inflated to their 2020 monetary equivalents based on the Australian Bureau of Statistics (ABS) Consumer Price Index (Health group) [24] (Table 2) and then employed within a set of arithmetic calculations involving the different proportions of the uptake rate and a 1% reduction in cost categories, individually, corresponding to each 1% decline in risk of disease.

Typically, the costs of disease are broken down into direct (i.e., healthcare-related) and indirect (i.e., productivity- and mortality-related) categories. As per the AIHW report [23], methodologies for measuring indirect costs are contentious and at an early stage of development. As such, the AIHW has decided to focus on the analysis of direct health system costs in the Disease Costs and Impact Study and to use, where appropriate, more direct measures of disease impact in health status terms, rather than estimates of indirect costs. Saving estimates of the cancer-related productivity losses and associated costs in Australia were thus not included in the present analysis.

### 2.5. Discounted Rate

The discount rate refers to the interest rate used to determine the present value of future monetary figures [25]. Following the methodology that we have outlined recently [15], a real discount rate of 7% was applied to the sum of savings in CRC and total cancer costs, separately, to assess the discounted value of whole grain intake over the longer term, using the net present value equation:

$$Discounted\;cost\;savings=savings\;at\;year\;0\;\times \;\frac{1}{{\left(1+r\right)}^{n}}$$

where *r* = real discount rate (7%) and *n* = years into the future. The conservative discount rate of 7%, which has been used across Australian jurisdictions [26], was applied to present day savings in cancer-related healthcare costs at five-year increments for a 20-year period after 2020 (year 0), including 0–4 years (very pessimistic), 5–9 years (pessimistic), 10–14 years (optimistic), and 15–19 years (universal).

## 3. Results

Table 3 and Table 4 summarize the potential savings in the annual direct healthcare costs associated with the reductions in relative risk of CRC and total cancer mortality, respectively, when whole grain intakes are increased from the current median level of 21 g/day to the DTI level of 48 g across proportions of the Australian adult population. Under the very pessimistic scenario, assuming a 5% uptake rate over the short term, our analysis predicted total healthcare savings equal to AUD 1.9 (95% CI 1.2–2.4) million in CRC cost and AUD 20.3 (95% CI 12.2–27.0) million in total cancer cost annually. With a 15% uptake rate over the short-to-medium-term, the pessimistic scenario showed savings of AUD 5.6 (95% CI 3.6–7.2) million for CRC and AUD 60.8 (95% CI 36.5–81.0) million for total cancer costs. The optimistic scenario, which assumed a 50% uptake rate and a medium-to-long-term effect, predicted savings of AUD 18.6 (95% CI 12.0–24.1) million for CRC and AUD 202.6 (95% CI 121.5–270.1) million for total cancer costs. Lastly, under the universal scenario, assuming a 100% uptake rate and a best-case, long-term estimate of potential savings with the targeted increase in whole grain intake, our analysis predicted total annual healthcare savings of AUD 37.2 (95% CI 24.1–48.1) million and AUD 405.1 (95% CI 243.1–540.1) million in avoided CRC and total cancer costs, respectively.

Using a 7% discount rate, as per the Australian government’s recommendations [26], Table 5 summarizes the monetary figures associated with the percent reduction in cancer risk following the 48 g DTI of whole grains over a 20-year timeframe, where the total discounted savings over a 20 year period was shown to range between AUD 21.1 (95% CI 13.6–27.3) million and AUD 421.4 (95% CI 272.7–545.3) million for CRC and between AUD 229.6 (95% CI 137.8–306.1) million and AUD 4592.1 (95% CI 2755.2–6122.8) million for total cancer. On the other hand, the sum of total incremental healthcare cost savings with adoption of each of the 4 scenarios every 5 years was estimated at AUD 126.2 (95% CI 81.6–163.3) million for CRC and AUD 1374.8 (95% CI 824.9–1833.1) million for total cancer.

## 4. Discussion

The resulting annual healthcare cost savings for total cancers of AUD 405 million, including AUD 37 million for CRC, equate to a possible 4.5% and 5% saving in annual healthcare cost, respectively. This research follows on from our recent analysis of T2DM and CVD [15] where AUD 1.4 billion in savings would be possible if more Australians met the 48 g whole grain DTI. Globally, by 2030, the direct medical and non-medical costs and income losses for cancer are projected to be USD 458 billion [27]. As a nation, Australia leads the world in CRC incidence, and the trends observed among younger adult diagnosis (<40 years) are concerning. Colon cancer has a long latency period, on average over 10 years, but can vary widely from 4 to 20 years [3]. This extended timeframe also provides an impetus to shift focus to slightly younger age groups for prevention messaging, improving dietary patterns, in addition to screening those over 50 years of age. For example, in 2019, AUD 10 million was reportedly spent just on the mass media campaign for CRC screening initiatives, equating to AUD 2500 per life saved, and a further prediction of 4330 lives to be saved over the following 40 years [28]. In comparison, over a 20-year timeframe, with increasing adoption of whole grain within diets (every five years), the expected saving due to prevention of total cancer is predicted to be AUD 1.4 billion with AUD 126 million attributable to CRC, a far greater saving in half the number of years.

Although Australians fall short of the 48 g whole grain daily target, total grain consumption on average has been stable between the NNPAS conducted in 1995 and then 2011–12, at 5.5 serves [29]. However, among adults over 19 years of age, there has been a decline in consumption of grain food serves from 5.6 to 5.5 per 10,000 kJ, the result of a 12% decrease in bread consumption (approximately 20 g less than 1995 [30]), with a 14% increase in consumption of other grains including rice, pasta and noodles, which would most probably be refined grain foods [31]. In the most recent survey, 66% of Australians consumed a median of 72 g of bread (approximately two average slices) on the day prior to the NNPAS interview [32]. For most (58%), this was white bread with mixed grain and wholemeal varieties accounting for 18% [32], noting that mixed grain breads rarely meet the definition of whole grain.

Whereas whole grain consumption sits below the target levels for adults (21 g) and children/adolescents (17 g), data for adolescents suggests their intake is lowest of all groups, at just 8.7 g/d, less than 20% of the 48 g DTI [16]. This is despite high energy needs, where core grain foods could be playing a role in providing energy, dietary fiber, and whole grains in addition to a range of other micronutrients. For example, wholemeal flour is naturally more nutrient dense than white flour with more calcium, chloride, fluoride, phosphorus, potassium, selenium, and zinc, twice as much magnesium and manganese, three times as much niacin, four times the folate, and eight times the amount of vitamin E [33]. As it is widely acknowledged that adolescent dietary patterns follow into adulthood, and poor habits may further predispose younger adults to earlier disease onset into the future, prevention strategies focused clearly on those food groups with the greatest modifiable effect need to be emphasized more clearly in dietary guidance at all stages of the lifecycle.

Although there are a number of meta-analyses examining CRC [9,10,11,12] this analysis utilized Aune et al. (2011) [9], as this represented a somewhat conservative view of risk reduction, 5.1% (95% CI 3.3–6.6) for the 27 g whole grain gap, compared to Reynolds et al. 2019 [12] with 5.4% (95% CI 1.8–9), indicating a potential larger saving at the higher end of the confidence interval from the latter. For a comparison of risk reduction results provided by the available meta-analyses, see Table A1 (Appendix A). As this study involved an etiological question, systematic reviews of level II evidence, such as case-control or prospective cohort studies, were identified as these provide more data than individual studies and the meta-analyses serves to increase the precision of the overall results and reduces the likelihood that results have occurred by chance.

Compared with biannual screening, expecting Australians to change their dietary pattern to adopt whole grain may be ambitious. In our earlier research, we acknowledged that a 100% adoption of the 48 g DTI across the entire population may not be possible due to specific dietary restrictions related to gluten containing grains and other special dietary requirements [15]. Unlike our earlier publication regarding CVD and T2DM, where we utilized healthcare costs and productivity loss savings [15], the data for productivity loss was not available from the AIHW for total cancer or CRC. For this reason, the results of the two publications cannot be directly compared or added together. Others have estimated the present value of lifetime income (PVLI) for men at AUD 2.9 billion and women at AUD 1 billion for total cancer, the most common and most costly cause of death for both genders based on 2003 data [34]. These figures indicate a likelihood of even higher cost savings for improving dietary related factors for both total cancer and CRC. 

## 5. Conclusions

There is a compelling case for prevention strategies for cancer, but particularly where there are a range of modifiable dietary factors; such is the case for CRC. From this analysis, there are substantial healthcare cost-savings of AUD 37.2 million for CRC on its own and AUD 405.1 million, for total cancer with an increasing proportion of Australians meeting the 48 g whole grain DTI. CRC alone represents 9% of the overall possible healthcare cost savings for total cancers, an outcome that could be achieved by directing more Australians to consume the target amount of whole grain. Although there are a wide range of core whole grain options, wholemeal bread, whole grain breakfast cereals and crackers can be simply accommodated within diets and a regular inclusion of these provides sufficient whole grain to reach the DTI of 48 g. Yet, in Australia, where disease rates for CRC are the highest in the world, the focus is almost solely on screening those >50 years of age. While this is undoubtedly an effective strategy, a trend towards younger people being diagnosed may thwart screening efforts over time. Prevention through simple dietary modification could be playing a more prominent role through very specific guidance towards whole grain food choices and greater support of promotional campaigns aligned with food-based dietary guidelines.

## Figures and Tables

**Table 1:** Summary of the cost-of-illness analysis input parameters and corresponding references.

Parameter	Men and Women	References
Current whole grain intake, g/day	21	Galea et al. [16]
Target whole grain intake, g/day	48	Griffiths et al., Chen et al., Zong et al. [18,19,20]
Difference, g/day	27	
Uptake rate (proportions of prospective consumers) ^1^	Very pessimistic (5%), Pessimistic (15%) Optimistic (50%), Universal (100%)	Estimates
Colorectal cancer risk		
Relative risk (95% CI) per 90 g/day increase in whole grain intake, no. of studies	0.83 (0.78–0.89), *n* = 6	Aune et al. [9]
% Risk reduction (95% CI) per 27 g/day ^2^	−5.1% (3.3–6.6)	
Total cancer mortality risk		
Relative risk (95% CI) per 90 g/day increase in whole grain intake, no. of studies	0.85 (0.80–0.91), *n* = 6	Aune et al. [21]
% Risk reduction (95% CI) per 27 g/day ^2^	–4.5% (2.7–6.0)	

^1^ Proportions of the Australian adult population (≥20 years) who would increase their whole grain consumption to the recommended (target) level of 48 g/day over the short term (very pessimistic), short-to-medium term (pessimistic), medium-to-long term (optimistic), and long term (universal). ^2^ Percent risk reduction (95% CI) per 27 g/day was calculated based on the summary relative risk (95% CI) values per 90 g/day by Aune et al. [9,21] assuming a linear relationship.

**Table 2:** Summary of colorectal cancer and all cancers direct health expenditures (AUD million) in Australian adults (age 20 and up) ^1^.

	Colorectal Cancer		Total Cancers	
	2015–16	2020 ^2^	2015–16	2020 ^2^
Direct health expenditure				
Allied health and other services	0.3	0.3	5.0	5.7
General practitioner services	13.2	15.0	303.1	345.0
Medical imaging	2.9	3.3	93.6	106.5
Pathology	3.3	3.8	137.4	156.5
Pharmaceutical benefits scheme	113.8	129.6	1285.1	1462.8
Private hospital services	195.2	222.2	2318.0	2638.5
Public hospital admitted patient	168.6	191.9	2103.8	2394.7
Public hospital emergency department	0.5	0.6	28.9	32.9
Public hospital outpatient	102.6	116.8	949.0	1080.2
Specialist services	39.9	45.4	684.8	779.5
All direct health expenditure	640.3	728.9	7908.8	9002.3

Abbreviations: AUD, Australian dollar. ^1^ From the Australian Institute of Health and Welfare (AIHW) disease expenditure database (2015-16) [23]. ^2^ Current dollars based on adjustment of inflation rates according to the Australian Bureau of Statistics (ABS) Consumer Price Index (Health group) [24].

**Table 3:** Potential annual savings in direct health expenditures of colorectal cancer in Australian adults (≥20 years) from whole grain intakes (AUD million) ^1^.

	Scenario			
	Very Pessimistic	Pessimistic	Optimistic	Universal
Direct health expenditure savings				
Allied health and other services	<0.01 (<0.01 -< 0.01)	<0.01 (<0.01 -< 0.01)	0.01 (0.01–0.01)	0.02 (0.01–0.02)
General practitioner services	0.04 (0.02–0.05)	0.11 (0.07–0.15)	0.38 (0.25–0.50)	0.77 (0.50–0.99)
Medical imaging	0.01 (0.01–0.01)	0.02 (0.02–0.03)	0.08 (0.05–0.11)	0.17 (0.11–0.22)
Pathology	0.01 (0.01–0.01)	0.03 (0.02–0.04)	0.10 (0.06–0.13)	0.19 (0.13–0.25)
Pharmaceutical benefits scheme	0.33 (0.21–0.43)	0.99 (0.64–1.28)	3.30 (2.14–4.28)	6.61 (4.28–8.55)
Private hospital services	0.57 (0.37–0.73)	1.70 (1.10–2.20)	5.67 (3.67–7.33)	11.33 (7.33–14.66)
Public hospital admitted patient	0.49 (0.32–0.63)	1.47 (0.95–1.90)	4.89 (3.17–6.33)	9.79 (6.33–12.66)
Public hospital emergency department	<0.01 (<0.01 -< 0.01)	<0.01 (<0.01–0.01)	0.02 (0.01–0.02)	0.03 (0.02–0.04)
Public hospital outpatient	0.30 (0.19–0.39)	0.89 (0.58–1.16)	2.98 (1.93–3.86)	5.96 (3.86–7.71)
Specialist services	0.12 (0.07–0.15)	0.35 (0.22–0.45)	1.16 (0.75–1.50)	2.32 (1.50–3.00)
All direct health savings	1.86 (1.20–2.41)	5.58 (3.61–7.22)	18.59 (12.03–24.05)	37.17 (24.05–48.11)

Abbreviations: AUD, Australian dollar. ^1^ Data (95% CI) are monetary savings following colorectal cancer risk reduction with whole grain intake (Table 1). The very pessimistic and pessimistic scenarios are, respectively, practical short term and short-to-medium-term estimates of expenditure savings that could follow when 5% and 15% of Australian adults (≥20 years) consume the daily target intake of whole grain. The optimistic scenario is a medium-to-long-term pragmatic estimate of potential savings when 50% of adults in Australia adopt the recommended level of whole grain. The universal scenario is a best-case long-term estimate of potential savings when 100% of Australian adults increase their intakes of whole grains.

**Table 4:** Potential annual savings in direct health expenditure of total cancer in Australian adults (≥20 years) from whole grain intakes (AUD million) ^1^.

	Scenario			
	Very Pessimistic	Pessimistic	Optimistic	Universal
Direct health expenditure savings				
Allied health and other services	<0.1 (<0.1 -< 0.1)	<0.1 (<0.1–0.1)	0.1 (0.1–0.2)	0.3 (0.2–0.3)
General practitioner services	0.8 (0.5–1.0)	2.3 (1.4–3.1)	7.8 (4.7–10.4)	15.5 (9.3–20.7)
Medical imaging	0.2 (0.1–0.3)	0.7 (0.4–1.0)	2.4 (1.4–3.2)	4.8 (2.9–6.4)
Pathology	0.4 (0.2–0.5)	1.1 (0.6–1.4)	3.5 (2.1–4.7)	7.0 (4.2–9.4)
Pharmaceutical benefits scheme	3.3 (2.0–4.4)	9.9 (5.9–13.2)	32.9 (19.7–43.9)	65.8 (39.5–87.8)
Private hospital services	5.9 (3.6–7.9)	17.8 (10.7–23.7)	59.4 (35.6–79.2)	118.7 (71.2–158.3)
Public hospital admitted patient	5.4 (3.2–7.2)	16.2 (9.7–21.6)	53.9 (32.3–71.8)	107.8 (64.7–143.7)
Public hospital emergency department	0.1 (<0.1–0.1)	0.2 (0.1–0.3)	0.7 (0.4–1.0)	1.5 (0.9–2.0)
Public hospital outpatient	2.4 (1.5–3.2)	7.3 (4.4–9.7)	24.3 (14.6–32.4)	48.6 (29.2–64.8)
Specialist services	1.8 (1.1–2.3)	5.3 (3.2–7.0)	17.5 (10.5–23.4)	35.1 (21.0–46.8)
All direct health savings	20.3 (12.2–27.0)	60.8 (36.5–81.0)	202.6 (121.5–270.1)	405.1 (243.1–540.1)

Abbreviations: AUD, Australian dollar. ^1^ Data (95% CI) are monetary savings following total cancers mortality risk reduction with whole grain intake (Table 1). The very pessimistic and pessimistic scenarios are, respectively, practical short term and short-to-medium-term estimates of expenditure savings that could follow when 5% and 15% of Australian adults (≥20 years) consume the daily target intake of whole grain. The optimistic scenario is a medium-to-long-term pragmatic estimate of potential savings when 50% of adults in Australia adopt the recommended level of whole grain. The universal scenario is a best-case long-term estimate of potential savings when 100% of Australian adults increase their intakes of whole grains.

**Table 5:** Sum of potential total discounted savings on direct health expenditures of total cancers and colorectal cancer in Australian adults (≥20 years) from whole grain intakes over short term and long-term periods (AUD million) ^1^.

	Scenario			
	Very Pessimistic	Pessimistic	Optimistic	Universal
	Total savings of years 0 to 4			
Colorectal cancer	**8.2 (5.3–10.6)**	24.5 (15.8–31.7)	81.5 (52.8–105.5)	163.1 (105.5–211.1)
Total cancer	**88.9 (53.3–118.5)**	266.6 (160.0–355.5)	888.6 (533.2–1184.8)	1777.3 (1066.4–2369.7)
	Total savings of years 5 to 9			
Colorectal cancer	5.8 (3.8–7.5)	**17.4 (11.3–22.6)**	58.1 (37.6–75.2)	116.3 (75.2–150.5)
Total cancer	63.4 (38.0–84.5)	**190.1 (114.0–253.4)**	633.3 (380.1–844.8)	1267.2 (760.3–1689.6)
	Total savings of years 10 to 14			
Colorectal cancer	4.1 (2.7–5.4)	12.4 (8.0–16.1)	**41.5 (26.8–53.6)**	82.9 (53.6–107.3)
Total cancer	45.2 (27.1–60.2)	135.5 (81.3–180.7)	**451.7 (271.0–602.3)**	903.5 (542.1–1204.6)
	Total savings of years 15 to 19			
Colorectal cancer	3.0 (1.9–3.8)	8.9 (5.7–11.5)	29.6 (19.1–38.2)	**59.1 (38.2–76.5)**
Total cancer	32.2 (19.3–42.9)	96.6 (58.0–128.8)	322.1 (193.2–429.4)	**644.2 (386.5–858.9)**
	Total discounted savings for each scenario (2020–2039)			
Colorectal cancer	**21.1 (13.6–27.3)**	**63.2 (40.9–81.8)**	**210.7 (136.3–272.7)**	**421.4 (272.7–545.3)**
Total cancer	**229.6 (137.8–306.1)**	**688.8 (413.3–918.4)**	**2296.0 (1377.6–3061.4)**	**4592.1 (2755.2–6122.8)**
	Total incremental discounted savings: adoption of each scenario every 5 years (2020–2039)			
Colorectal cancer	-	-	-	**126.2 (81.6–163.3)**
Total cancer	-	-	-	**1374.8 (824.9–1833.1)**

Abbreviations: AUD, Australian dollar. ^1^ Data (95% CI) are monetary savings following total cancer and colorectal cancer risk reductions with whole grain intake. The very pessimistic and pessimistic scenarios are, respectively, practical short term and short-to-medium-term estimates of expenditure savings that could follow when 5% and 15% of Australian adults (≥20 years) consume the daily target level of whole grain. The optimistic scenario is a medium-to-long-term pragmatic estimate of potential savings when 50% of adults in Australia adopt the recommended level of whole grain. The universal scenario is a best-case long-term estimate of potential savings when 100% of Australian adults increase their intakes of whole grains.

## Data Availability

All data for this study is contained within the article.

## Appendix A

**Table A1:** Evidence from meta-analyses of cohorts or case-control studies associating different doses of whole grain intake with the relative risk or odds ratio of colorectal cancer and total cancer mortality ^1^.

Meta-Analysis	No. of Studies Included	Relative Risk or Odds Ratio (95% CI)	% Reduction in Relative Risk or Odds Ratio (95% CI) per 27 g/day ^2^
Whole grain and risk of colorectal cancer			
Aune et al., 2011 [9]	6	0.83 (0.78–0.89) per 90 g/day	−5.1% (3.3–6.6)
Vieira et al., 2017 [10]	6	0.83 (0.79–0.89) per 90 g/day	−5.1% (3.3–6.3)
Schwingshackl et al., 2018 [11]	9	0.95 (0.93–0.97) per 30 g/day	−4.5% (2.7–6.3)
Reynolds et al., 2019 [12]	8	0.97 (0.95–0.99) per 15 g/day	−5.4% (1.8–9.0)
Whole grain and risk of total cancer mortality			
Aune et al., 2016 [21]	6	0.85 (0.80–0.91) per 90 g/day	−4.5% (2.7–6.0)
Benisi-Kohansel et al., 2016 [35]	3	0.90 (0.83–0.98) per 90 g/day	−3.0% (0.6–5.1)
Chen et al., 2016 [19]	6	0.82 (0.69–0.86) per 50 g/day	−9.7% (7.6–16.7)
Wei et al., 2016 [36]	7	0.91 (0.84–0.98) per 90 g/day	−2.7% (0.6–4.8)
Zong et al., 2016 [20]	10	0.80 (0.72–0.89) per 70 g/day	−7.7% (4.2–10.8)
		0.85 (0.76–0.94) per 50 g/day	−8.1% (3.2–13.0)
		0.89 (0.79–0.99) per 30 g/day	−9.9% (0.9–18.9)
		0.96 (0.91–1.01) per 10 g/day	−10.8% (0–24.3)
Zhang et al., 2018 [37]	14	0.97 (0.95–0.99) per 28 g/day	−2.9% (1.0–4.8)
Reynolds et al., 2019 [12]	7	0.95 (0.93–0.97) per 15 g/day	−9.0% (5.4–12.6)

^1^ Adapted from Gaesser 2020 [7]. ^2^ Calculated based on relative risk or odds ratio values by each of the corresponding references assuming a linear relationship.
